# Fixed drug eruption to propofol: Case series and clinical implications 

**DOI:** 10.5414/ALX02645E

**Published:** 2026-09-25

**Authors:** Carole Guillet, Elisabeth Gössinger, Lukas Joerg, Barbara Ballmer-Weber, Felicitas Bellutti-Enders, Valentina N.S. Hongler, Hazem A. Juratli, Karin Hartmann, Thierry Girard, Anne Leuppi-Taegtmeyer, Kathrin Scherer, Minaya Beigi, Esther Helen Steveling-Klein

**Affiliations:** 1 Division of Allergy, Department of Dermatology,; 2 Department of Biomedicine, University Hospital Basel and University of Basel, Basel,; 3 Faculty of Medicine, University Basel, Basel,; 4 Department of Dermatology, University Hospital Zurich, Zurich,; 5 Faculty of Medicine, University of Zurich, Zurich,; 6 Division of Allergology and Clinical Immunology, Department of Pulmonary Medicine, Allergology and Clinical Immunology, Inselspital, Bern University Hospital, University of Bern, Bern,; 7 Division of Allergy, Clinic for Dermatology and Allergology, Kantonsspital St. Gallen, St. Gallen,; 8 Allergy Unit, University Children’s Hospital Basel, Basel,; 9 Department of Dermatology, Universityhospital Basel, Basel,; 10 Department of Pathology, University Hospital Basel, Basel,; 11 Department of Clinical Research, University Hospital Basel and University of Basel, Basel,; 12 Clinic for Anaesthesia, Intermediate Care, Prehospital Emergency Medicine and Pain Therapy, University Hospital Basel and University of Basel, Basel,; 13 Department of Patient Safety, Medical Directorate, University Hospital Basel, Basel,; 14 Clinical Pharmacology and Clinical Pharmacy Service, Cantonal Hospital Baselland, Liestal, and; 15 Dermatology and Allergology, Cantonal Hospital Aarau, Aarau, Switzerland; *Equally contributing first and equally contributing last authors.

**Keywords:** fixed-drug eruption, propofol, drug provocation testing

## Abstract

Background: Fixed drug eruption (FDE) is a type IV delayed hypersensitivity reaction characterized by recurrent lesions at the same site upon drug re-exposure. Propofol-induced FDE is extremely rare. Case Report: We report four cases of FDE following propofol administration, presenting with erythematous or bullous lesions at fixed sites, sometimes with mucosal involvement. Standard skin tests and lymphocyte transformation tests were frequently negative, particularly when performed outside previously affected areas. Diagnosis was confirmed by controlled intravenous provocation in one adult case. Histology supported the diagnosis when available. Discussion: FDE to propofol is likely under-recognized due to delayed onset of lesions. Diagnostic testing can be challenging, and controlled re-exposure may be required. Awareness of this rare reaction is important given propofol’s widespread use and limited alternative anaesthetic options. Reporting such cases improves recognition and guides safe management.

## Introduction 

Fixed drug eruption (FDE) is a type IV delayed hypersensitivity reaction characterized by recurrence of lesions at the same site upon drug re-exposure. Propofol, a widely used intravenous anesthetic, is rarely associated with perioperative anaphylaxis [1, 2]. FDE due to propofol is uncommon, with only two cases reported [3, 4]. We present the clinical features of propofol-induced FDE and discuss diagnostic steps, including provocation testing using a proposed protocol. 

## Case 1 

A 57-year-old woman was referred for allergy workup after developing targetoid macules and a focal bulla on the chest, back, and proximal extremities 3 days after propofol sedation (Figure 1, left). Mucous membranes were spared. Histology showed a lichenoid dermatitis, consistent with a drug eruption. Similar lesions had occurred three years earlier after general anesthesia for breast cancer surgery. At that time, a skin biopsy indicated a drug eruption, and the lesions resolved with post-inflammatory hyperpigmentation following topical and systemic steroid treatment. Allergy evaluation, including skin prick and intradermal testing, was performed according to the guidelines of the European Network on Drug Allergy (ENDA) and the European Academy of Allergy and Clinical Immunology (EAACI) [5]. 

Propofol was initially only tested on the forearm, using a concentration of 10 mg/mL for skin prick testing and 1 mg/mL and 10 mg/mL for intradermal testing. All test results were negative at 30 minutes, as well as after 24 and 48 hours. Two cytokine-based lymphocyte transformation testings (Cyto-LTT), performed 8 months apart, showed no signs of T-cell-mediated hypersensitivity reaction to propofol. The test was performed by Drug Reaction Analysis Center (ADR-AC), Bern, Switzerland according to previously established protocols [6]. 

The patient subsequently underwent a titrated intravenous provocation starting with 5 mg propofol with subsequent increments of 10 mg every 20 minutes up to a cumulative dose of 95 mg under close monitoring, to minimize sedative effects (Table 1). Within 24 hours, erythematous pruritic macules reappeared at previously affected sites, confirming fixed drug eruption (Figure 1, right). Histology supported the diagnosis revealing a vacuolar interface dermatitis with lymphocytic exocytosis, intraepidermal presentation of apoptotic keratinocytes and a perivascular accentuated lymphohistiocytic infiltrate with admixture of diffusely distributed eosinophilic granulocytes and evidence of melanin pigment incontinence. Routine laboratory parameters were within normal limits. A repeated intradermal test with propofol (1 mg/mL and 10 mg/mL) performed in loco on the back yielded a positive reaction at 24 hours (Figure 2). 

## Case 2 

An 8-year-old boy with a history of glioma presented with a persistent 3 × 4 cm erythematous-livid plaque on the right chest. The lesion had first appeared at age 5 after repeated surgical procedures. It darkened within 24 hours post-intervention and healed with desquamation; a hyperpigmented macule remained. Chart review identified paracetamol and propofol as the only potential elicitors. Paracetamol challenge was negative. Due to the patients’s age, intradermal testing and intravenous provocation were not performed. Later procedures avoiding propofol and using sevoflurane were uneventful. 

## Case 3 

A 49-year-old man developed bullous lesions on the palms and soles with mild oral involvement (Figure 3) 3 days after cardioversion under propofol sedation. He had previously tolerated propofol during cardiac catheterization. No other drugs were introduced. The eruption resolved with systemic steroids. Patch testing was negative, Cyto-LTT, also performed at the ADR-AC, Bern, Switzerland [6], using a standardized protocol including parallel testing in autologous plasma and AB serum, demonstrated elevated interferon-γ, granzyme B, and granulysin levels, especially in AB serum, indicating a T-cell-mediated hypersensitivity reaction to propofol. A skin biopsy was not performed. 

## Case 4 

A 35-year-old woman with a history of multiple abdominal surgeries consistently developed sharply demarcated erythematous lesions several hours after each surgical procedure. The eruptions progressed to blister formation within hours and resolved spontaneously over days to weeks. All lesions consistently recurred at the same locations: the left hand, the genital area, and the oral mucosa. 

A total of 3 reactions were documented at our institution, with additional similar episodes reported by the patient following earlier surgeries performed externally. The interval between the first 2 documented reactions was ~ 6 months, 12 months between the second and third, and 7 months between the last reaction and allergological evaluation. Propofol had been administered prior to each of these reactions and was the only consistently used drug across all procedures. 

Patch and intradermal testing with propofol outside previously affected skin areas were negative. Based on the characteristic clinical presentation and the reproducible temporal association, a fixed bullous drug eruption to propofol was diagnosed. Histological examination was not performed. After avoidance of propofol in a subsequent surgery, no further reactions occurred. 

## Discussion 

We report 4 cases of FDE following propofol administration. To date, FDE or type IV hypersensitivity to propofol is rarely described. The delayed onset of lesions – often after discharge – may contribute to overlook the disease. Given propofol’s widespread use, such reactions may be overlooked. Diagnostic testing is challenging. In our first case, both intradermal testing outside the lesion and the Cyto-LTT were negative. However, intradermal testing performed in a previously affected area was positive, thereby supporting the diagnosis in conjunction with the provocation test. In case 3, Cyto-LTT yielded a positive reaction to propofol, suggesting that this assay may represent a useful adjunctive diagnostic tool in selected cases. 

Taken together, our findings – negative skin testing in non-affected skin, positive intradermal testing in previously involved sites, and a positive Cyto-LTT in 1 case – suggest that the diagnostic yield of these methods may vary depending on test location and modality. This highlights the limited sensitivity of skin testing in FDE, particularly when performed on uninvolved skin. The potential immunomodulatory effects of propofol may further contribute to reduced test sensitivity [7]. 

In case 3, the involvement of palms and soles with bullous lesions represents an unusual distribution for fixed drug eruption; however, atypical palmoplantar and bullous variants have been described, particularly in more extensive reactions. In the present case, the early timing of clinical documentation likely accounts for the pronounced erythematous appearance, while subsequent evolution to post-inflammatory hyperpigmentation was consistent with fixed drug eruption. 

In cases with no contraindication for provocation and negative skin- or invitro-testing, controlled intravenous re-exposure may clarify the diagnosis. For suspected FDE or other delayed-type hypersensitivity reactions to propofol, we propose a stepwise protocol (Table 1). Unlike standard DPT recommendations, we used repeated doses of 10 mg every 20 minutes rather than dose increments. This approach aimed to avoid sedative effects while achieving a cumulative dose sufficient to elicit a delayed response. 

From an anesthesiologic perspective, hypersensitivity to propofol is highly relevant. In many countries, propofol is the primary induction agent, as thiopentone is no longer available and etomidate is limited due to adrenal suppression [8]. Alternative agents such as dexmedetomidine, ketamine, or remimazolam remain limited, highlighting the importance of confirming true hypersensitivity before excluding propofol use. The WHO pharmacovigilance database lists ten reports of “fixed eruption” linked to propofol, representing only 0.026% of 38,335 entries [9]. However, given the high underreporting rate – estimated at 94% in a systematic review – the true number is likely higher [10]. Case reports thus remain essential to improve recognition and guide risk assessment of rare adverse drug reactions like propofol-induced FDE. 

## Declaration of AI and AI-assisted technologies in the writing process 

During the preparation of this work the author(s) used ChatGPT powered by GPT-4 (OpenAI, accessed via chat.openai.com in July 2025) in order to support the formulation and refinement of the manuscript. After using this tool/service, the author(s) reviewed and edited the content as needed and take(s) full responsibility for the content of the publication. 

## Ethics statement 

Informed consent to publish the case reports and all accompanying visual elements was obtained from all patients involved. In the case of the 8 year old patient informed consent was obtained from his legal guardians. 

## Authors’ contributions 

This manuscript represents an inter-university collaboration across the University Hospitals of Zurich, Basel, Bern, St. Gallen and Aarau, which is why multiple authors were involved. Only those with significant contributions are listed here: 

Carole Guillet: Patient recruitment, clinical assessment, manuscript drafting and revision, final approval. 

Elisabeth Gössinger: Patient recruitment, clinical assessment, manuscript drafting and revision, final approval. 

Lukas Joerg: Patient recruitment, clinical assessment, manuscript drafting and revision, final approval. 

Barbara Ballmer-Weber: Patient recruitment, clinical assessment, manuscript drafting and revision, final approval. 

Felicitas Bellutti-Enders: Patient recruitment, clinical assessment, manuscript drafting and revision, final approval. 

Valentina N. S. Hongler: Patient recruitment, clinical assessment, manuscript drafting and revision, final approval. 

Hazem A. Juratli: Patho-histological diagnostic, manuscript drafting and revision, final approval. 

Karin Hartmann: Conceptualization of the study, manuscript drafting and revision, final approval. 

Thierry Girard: Conceptualization of the study, manuscript drafting and revision, final approval. 

Anne Leuppi-Taegtmeyer: Conceptualization of the study, manuscript drafting and revision, final approval. 

Kathrin Scherer: Conceptualization of the study, manuscript drafting and revision, final approval. 

Minaya Beigi: Patient recruitment and clinical assessment, conceptualization of the study, manuscript drafting and revision, final approval. 

Esther Helen Steveling-Klein: Patient recruitment and clinical assessment, conceptualization of the study, manuscript drafting and revision, final approval. 

## Funding 

KH received research funding from the Swiss National Science Foundation (SNSF; grant 310030_207705), the Swiss Cancer Research Foundation (gant KFS-5979-08-2023) and the EU-H2020-MSCA-COFUND EURIdoc programme (No. 101034170). 

## Conflict of interest 

KH received honoraria for lectures, consulting and/or advisory boards from ALK, Allergopharma, Almirall, BioCryst, Blueprint Medicines, Cogent, Galderma, KalVista, Leo, Menarini, Novartis, Pfizer, Sanofi, Takeda and Thermo Fisher. 

CG received honoraria for lectures, consulting and/or advisory boards from Sanofi. 

ESK received honoraria for lectures, consulting and/or advisory boards from ALK, Leo pharma, GSK, Vifor, Pierre Fabre, Dr. Falk Pharma, Allergopharma 

BBW received honoraria for lectures or advisory boards from ALK, Stallergenes, Blueprint Medicines, MSD, Novartis, Sanofi, Takeda and Thermo Fisher. 

No COI for all other authors. 

**Figure 1.**
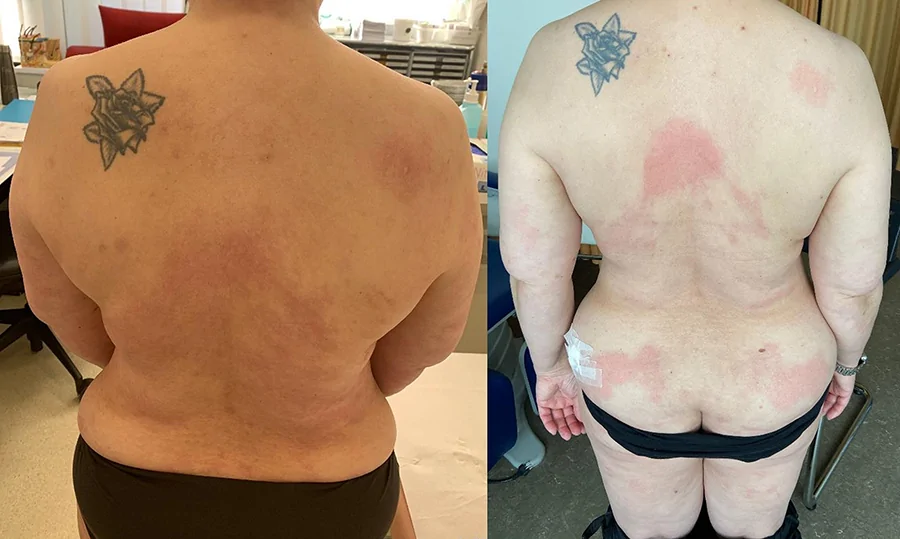
Left: 57-year-old woman with multilocular fixed drug eruption after propofol sedation for endoscopic examination. Picture 5 days after onset of drug eruption. Right: Two days after propofol provocation. Erythematous macules reappeared in loco on the torso and proximal extremities.

**Table 1.** Propofol provocation for delayed-type hypersensitivity.

**Provocation step**	**Propofol (mg)**	**Cumulative dose (mg)**	**Amount (mL)**	**Time (minutes)**
1	5	5	0.5 mL	15
2	10	15	1.0 mL	35
3	10	25	1.0 mL	55
4	10	35	1.0 mL	75
5	10	45	1.0 mL	95
6	10	55	1.0 mL	115
7	10	65	1.0 mL	135
8	10	75	1.0 mL	155
9	10	85	1.0 mL	175
10	10	95	1.0 mL	195

**Figure 2.**
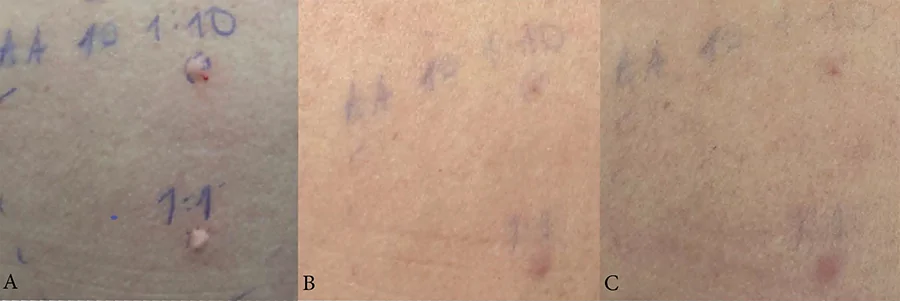
Intradermal skin test with propofol 10 mg/mL, 1 : 10 (top), 1 : 1 dilution (undiluted, bottom). A: Immediately after testing. B: Read-out after 24 hours: Erythema and papule in the 1 : 1 dilution. C: Read-out after 48 hours: Erythema and hyperpigmentation in the 1 : 1 solution.

**Figure 3.**
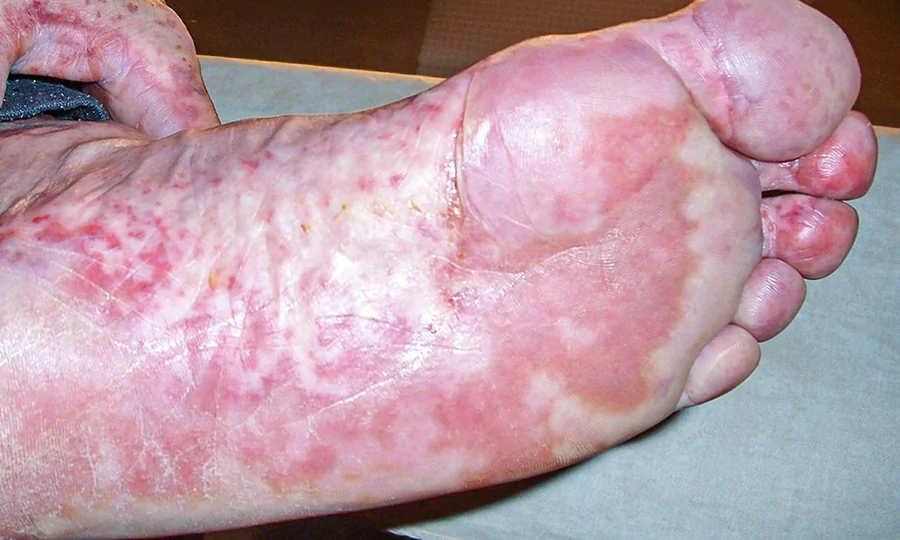
Soles of a 49-year-old man 3 days after cardioversion using propofol. Bullous erythematous targetoid skin lesions appeared on soles and palms.
